# Ultrasonographic and serum biomarkers for diagnosis of biliary atresia

**DOI:** 10.1007/s00383-026-06484-6

**Published:** 2026-06-18

**Authors:** Betina Carla Bertrand Simões, Ivonete Siviero, Claudia Renata Rezende Penna, Elazir Barbosa Mota Di Puglia, Sandra Valéria Coelho da Silva, José Eduardo Ferreira Manso

**Affiliations:** 1 Instituto de Pediatria e Puericultura Martagão Gesteira (IPPMG), Federal University of Rio de Janeiro, Rio de Janeiro, Brazil; 2 Department of Surgery, Federal University of Rio de Janeiro, Rio de Janeiro, Brazil

**Keywords:** Biliary atresia, Cholestasis, Gamma-glutamyltransferase, Ultrasonography, Biomarkers

## Abstract

**Purpose:**

Biliary atresia (BA) is the main cause of neonatal cholestasis and the leading indication for pediatric liver transplantation. Early diagnosis is essential for timely intervention and improved outcomes. This study evaluated accessible biomarkers for the diagnosis of BA, including serum gamma-glutamyl transferase (GGT) and ultrasonographic (US) parameters.

**Methods:**

This retrospective study included infants with suspected BA treated between 2014 and 2024, at a tertiary pediatric center in Brazil. Serum GGT levels and US parameters were analyzed and the diagnosis was confirmed by surgical findings. Diagnostic accuracy was assessed using a ROC curve and logistic regression.

**Results:**

Sixty-seven infants were included, of whom 45 (67.2%) had confirmed BA. Median serum GGT levels were higher in BA cases (607 U/L) than in non-BA cases (243 U/L). ROC analysis identified a cutoff value of 311 U/L, with a sensitivity of 82.2% and specificity of 68.2%. US parameters were present in all BA cases, with 100% sensitivity and 86% specificity (*p* < 0.001). Combining GGT with abnormal US findings increased specificity to 95.5%.

**Conclusion:**

Ultrasonographic parameters alone, as well as their combination with elevated serum GGT levels, demonstrated high diagnostic value for identification of biliary atresia in infants with neonatal cholestasis.

## Introduction

Biliary atresia (BA) is the leading surgical cause of neonatal cholestasis and the most common indication for pediatric liver transplantation [1, 2]. It is characterized by progressive fibroinflammatory obliteration of the extrahepatic bile ducts, resulting in cholestasis, biliary cirrhosis, and eventually end-stage liver disease [3–5]. 

Despite its clinical relevance, the diagnosis of BA remains challenging because clinical, laboratory, and imaging findings often overlap with those of other causes of neonatal cholestasis [6]. Several diagnostic modalities have been proposed to improve diagnostic accuracy, including abdominal ultrasonography (US), serum gamma-glutamyl transferase (GGT) levels, hepatobiliary scintigraphy, magnetic resonance cholangiography, and liver biopsy. However, many of these methods are invasive, expensive, or require specialized expertise.

Abdominal US is widely available and non-invasive. Certain ultrasonographic findings, such as gallbladder abnormalities and the triangular cord sign, have been reported to be highly suggestive of BA [7–10]. In addition, elevated serum GGT levels—particularly values exceeding 300 U/L—are frequently observed in patients with BA and may serve as a useful adjunct diagnostic marker [7, 11]. 

Early diagnosis is critical for improving clinical outcomes. The definitive diagnosis is often established during surgical exploration, with or without intraoperative cholangiography, where fibro-obliteration of the extrahepatic bile ducts confirms the condition [1, 6]. Once confirmed, Kasai portoenterostomy is the treatment of choice and is significantly more effective when performed before 60 days of life [1, 2, 12]. Delayed surgical intervention reduces effective bile drainage due to progressive hepatic fibrosis and increases the risk of complications, prolonged hospitalization, higher healthcare costs, and earlier need for liver transplantation [13, 14]. 

Given the need for reliable, non-invasive, and accessible diagnostic strategies, the present study aimed to evaluate the diagnostic performance of specific ultrasonographic parameters and elevated serum GGT levels in differentiating BA from other causes of neonatal cholestasis in a tertiary pediatric referral center over a 10-year period.

## Methods

A retrospective observational study was conducted at a tertiary pediatric referral center in Brazil between January 2014 and January 2024. The institution is a regional referral center for the surgical management of biliary atresia (BA), receiving a high proportion of patients with suspected BA from other healthcare facilities. Ethical approval was obtained from the institutional research ethics committee.

All patients referred to our hospital for suspected cholestasis during this 10-year period were initially considered eligible for the study according to the predefined inclusion criteria (total of 80 patients). The inclusion criteria comprised infants presenting with cholestatic jaundice, defined as direct bilirubin levels greater than 20% of total bilirubin, associated with acholic or hypocholic stools.

Subsequently, 13 patients were excluded because ultrasonographic examinations, laboratory tests, or surgical procedures had been performed outside our institution. Eleven patients had already undergone imaging studies at other institutions and did not repeat the examinations before surgical exploration at our hospital, while two patients underwent surgical treatment at other centers by family choice.

These exclusions were adopted to ensure data standardization and reliability. The study depended on imaging and laboratory assessments performed according to standardized institutional protocols and interpreted by experienced professionals from our center, in addition to standardized surgical and histopathological confirmation.

All patients (67) underwent abdominal ultrasonography using a Toshiba Aplio 300 ultrasound system (Toshiba Medical Systems Corporation, Tokyo, Japan) equipped with high-frequency convex and linear transducers (6–14 MHz). All examinations were performed by an experienced pediatric radiologist, and the images were subsequently reviewed by another radiologist to ensure diagnostic reliability. In cases of disagreement in image interpretation, a third specialist was consulted for the final decision. Given the operator-dependent nature of ultrasonographic evaluation in BA, all examinations were performed according to standardized institutional protocols. Patients fasted for at least four hours prior to the examination.

Ultrasonographic findings were considered suggestive of BA when at least one of the following features was present: non-visualization of the gallbladder; gallbladder length < 1.5 cm with irregular margins or absence of the mucosal lining; or the visualization of the triangular cord sign, defined as an echogenic thickness > 2 mm anterior to the right portal vein or > 3 mm at the portal bifurcation [15]. Other sonographic features suggestive of BA were not evaluated in this study.

Serum gamma-glutamyl transferase (GGT) levels were measured in the institutional clinical laboratory. For analysis, the serum GGT value obtained to the ultrasonographic examination was selected. The institutional reference value for serum GGT was < 70 U/L.

The diagnosis of biliary atresia was confirmed by exploratory laparotomy, which allowed definitive diagnosis, anatomical classification according to Hartley et al. [5] and performance of Kasai portoenterostomy when feasible. In all cases, liver biopsy was performed for histopathological confirmation.

Eligibility for Kasai portoenterostomy was determined intraoperatively by the pediatric surgery team based on the anatomy of the extrahepatic biliary remnants and the accessibility of the porta hepatis allowing construction of the portoenterostomy.

Statistical analyses were performed using Microsoft Excel^®^ for data organization and R statistical software (R Foundation for Statistical Computing, Vienna, Austria). Continuous variables were described using medians and interquartile ranges due to non-parametric data distribution. The optimal GGT cutoff value was determined using receiver operating characteristic (ROC) curve analysis [16]. Diagnostic performance was assessed through sensitivity, specificity, predictive values, and logistic regression models. Associations were evaluated using Fisher’s exact test. A post hoc power analysis was performed to evaluate the statistical robustness of the sample.

## Results

All patients (total of 80 infants) fulfilling the study inclusion criteria were evaluated during the study period. Thirteen patients were excluded according to the predefined exclusion criteria, resulting in a final sample of 67 infants. Among these, 45 (67.2%) had confirmed BA, whereas 22 (32.8%) were diagnosed with other causes of neonatal cholestasis. These included 15 cases of neonatal unspecific hepatitis, five cases of bile duct hypoplasia (including two patients with Alagille syndrome), and two cases of choledochal cyst.

Because there are no standardized national epidemiological data regarding the incidence of BA in Brazil, the post hoc power analysis was based on the estimated prevalence reported in Western populations, approximately one case per 15,000–20,000 live births [4, 5], a post-hoc statistical power analysis was performed based on the study sample of 67 infants (45 with confirmed biliary atresia and 22 without the disease). The comparison between the observed proportions (67.16% vs. 32.84%) resulted in a Cohen’s effect size of *h* = 0.71, classified as moderate according to Cohen (2013) [17] Under these assumptions, the calculated statistical power was 98.2%, indicating a high probability of detecting a statistically significant difference between the groups.

All cases of BA were confirmed by surgical findings and histopathological analysis. Among the 45 patients with BA, Kasai portoenterostomy was performed in 28 patients (62.2%), while the procedure could not be performed in 17 patients (37.8%) due to inadequate access to the porta hepatis for portoenterostomy (advanced-stage liver disease).

According to the anatomical classification [5], two cases (4.4%) were classified as type I BA, five (11.1%) as type II, and 38 (84.4%) as type III. Regarding the clinical classification [18], 38 cases presented the isolated form of BA, 4 cases were identified as the embryonic form, and 3 cases were associated with cytomegalovirus (CMV) infection.

Age at diagnosis, defined as the age at surgical exploration, ranged from 3 to 36 weeks. The mean age at diagnosis was 92.5 days (approximately 13 weeks), with a median of 73 days (approximately 10 weeks). The mean age at ultrasonographic evaluation was 78 days (approximately 11 weeks), with a median of 63 days (approximately 9 weeks).

**Fig. 1:**
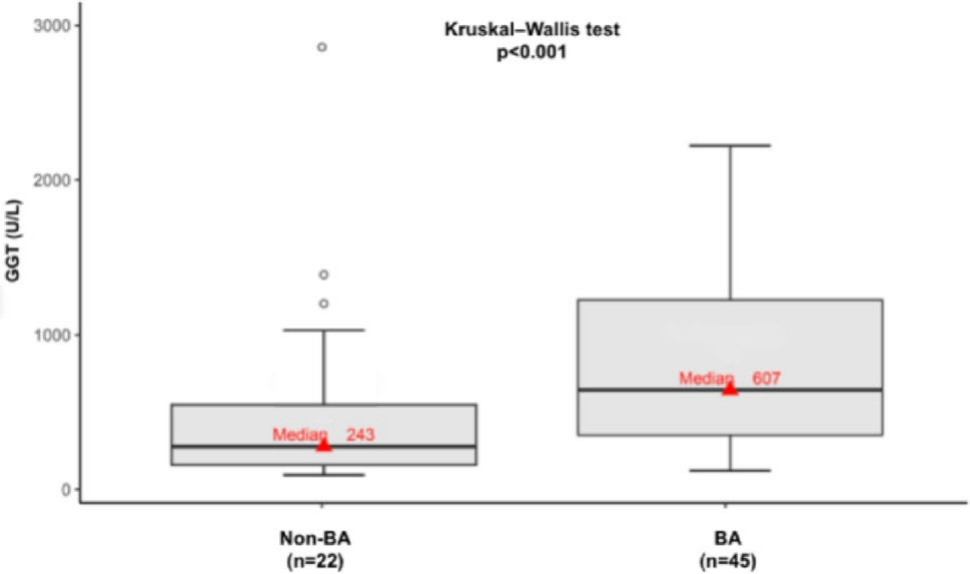
Boxplot of serum GGT levels in infants with biliary atresia (BA) and non-biliary atresia (non-BA). Median values are shown, and differences between groups were assessed using the Kruskal–Wallis test (*p* < 0.001)

The median serum GGT level among patients with BA was 607 U/L, compared with 243 U/L among patients without BA (Fig. 1).

**Fig. 2:**
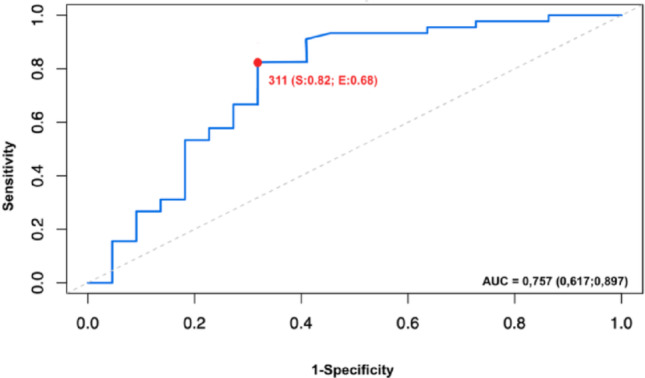
Receiver operating characteristic (ROC) curve for serum GGT levels in the diagnosis of BA. The optimal cutoff value was 311 U/L, with sensitivity of 82% and specificity of 68%. The area under the curve (AUC) was 0.757 (95% CI: 0.617–0.897)

The ROC curve analysis identified an optimal cutoff value of 311 U/L for serum GGT levels, yielding a sensitivity of 82.2% and specificity of 68.2%. The positive predictive value and negative predictive value were 84.1% and 65.2%, respectively (Fig. 2).

**Fig. 3:**
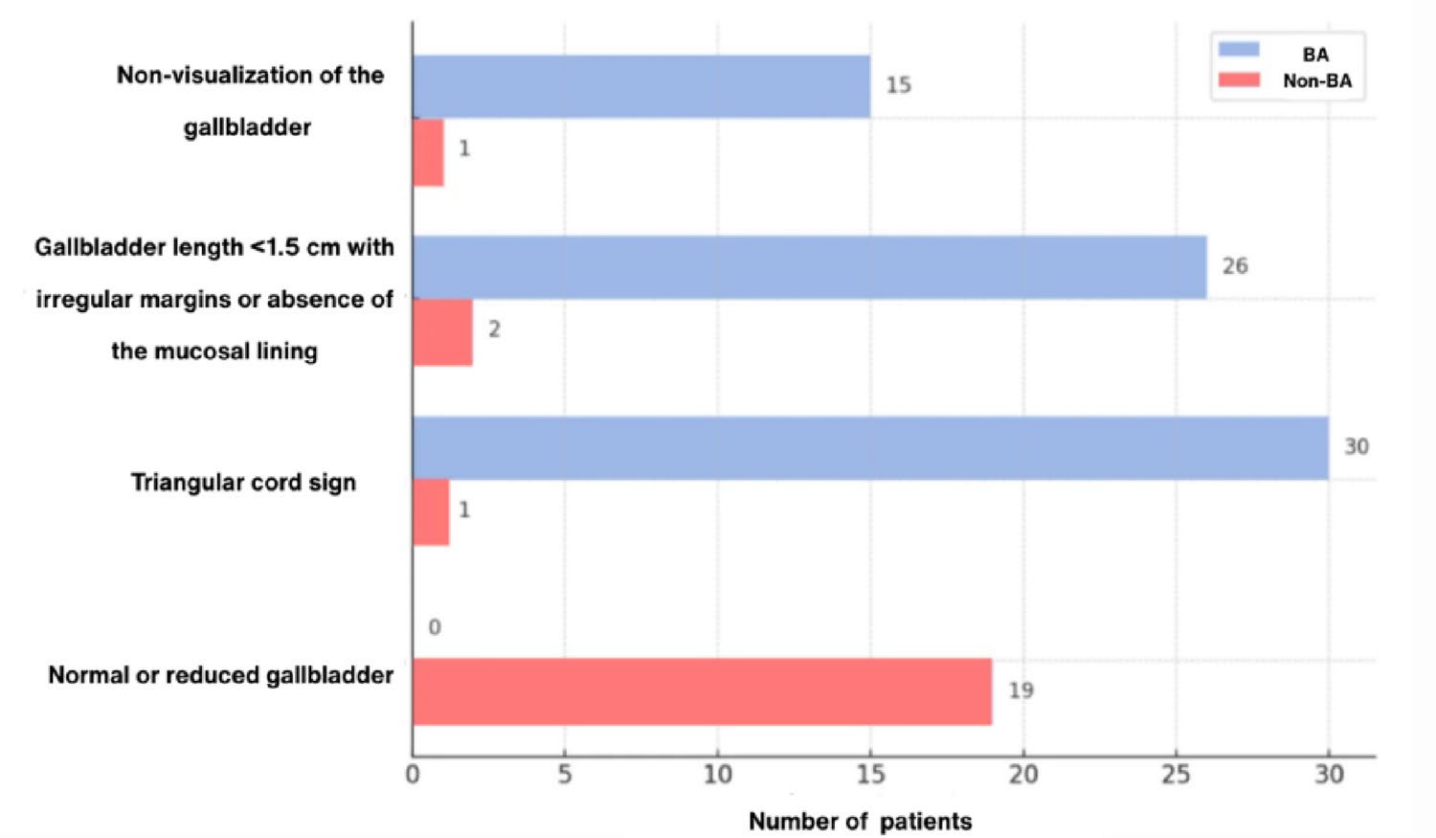
Distribution of ultrasonographic parameters in infants with biliary atresia (BA) and non-biliary atresia (non-BA)

The ultrasonographic parameters evaluated as biomarkers in this study were individually significantly associated with BA and included non-visualization of the gallbladder ( atretic) gallbladder), reduced gallbladder size (< 1.5 cm) with an irregular margins or absence of the mucosal lining, and the triangular cord sign (all *p* < 0.001) (Fig. 3).

US biomarkers were identified in 48 of 67 cases (71.6%), including all confirmed cases of BA. Three false-positive cases (19.9%) were observed, corresponding to two patients with bile duct hypoplasia and one with neonatal hepatitis. No false-negative cases were identified in this study population. In 19 cases (28.4%), US parameters were absent, all corresponding to non-BA cases. (Table 1) Ultrasonography demonstrated a sensitivity of 100% and a specificity of 86%, with an overall diagnostic accuracy of 96%.

**Table 1:**
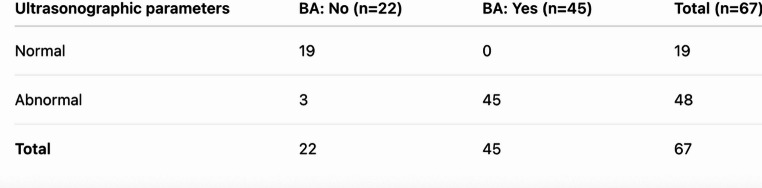
Diagnostic performance of ultrasonographic parameters for BA

Chi-square test with Yates’ correction and Fisher’s exact test: *p* < 0.0001; Odds ratio: infinity (95% confidence interval: 39.06–infinity).

The association between ultrasonographic biomarkers and BA was statistically significant (Fisher’s exact test: *p* < 0.0001).

**Fig. 4:**
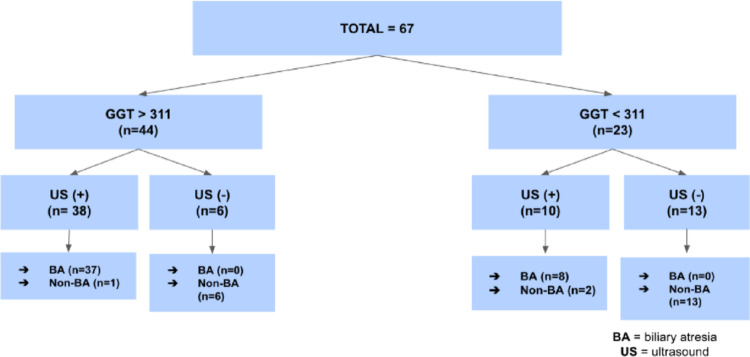
Illustrative diagram showing the distribution of cases according to serum GGT levels (> 311 U/L and < 311 U/L) and US findings. BA biliary atresia; GGT, gamma-glutamyl transferase; non-BA, non-biliary atresia; US (+), ultrasonography positive for biliary atresia; US (−), ultrasonography negative for biliary atresia

When elevated serum GGT levels (> 311 U/L) were combined with abnormal ultrasonographic findings (Fig. 4), diagnostic specificity increased to 95.5%, while maintaining a sensitivity of 82.2%. The odds ratio for the diagnosis of biliary atresia using the combined approach was 87.9 (95% CI: 11.3–4005.5; *p* < 0.001) (Table 2).

**Table 2:**
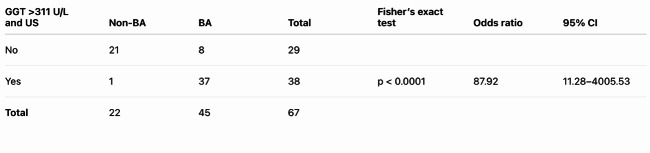
Association between GGT levels (> 311 U/L) and ultrasonographic findings

BA, biliary atresia; CI, confidence interval; GGT, gamma-glutamyl transferase; non-BA, non-biliary atresia; US (+), ultrasonography positive for biliary atresia

## Discussion

BA remains the leading cause of neonatal cholestasis and chronic liver disease in childhood, accounting for approximately 25–40% of cases [2]. In the present study, US biomarkers, alone or combined with elevated serum GGT levels, demonstrated high diagnostic performance in a tertiary referral cohort of infants with neonatal cholestasis. These simple and widely avaible findings reinforce the potential role of accessible and non-invasive diagnostic tools in supporting the evaluation and referral of patients with suspected BA. They also underscore the importance of strategies aimed at reducing diagnostic delay, as the mean age at diagnosis in our cohort was 13 weeks, a period already associated with poorer outcomes after Kasai portoenterostomy.

The diagnosis of BA was confirmed in 67.2% of the 67 infants included in the study, representing a higher proportion than reported in most neonatal cholestasis series [2]. These findings may reflect the tertiary referral profile of our institution; a university pediatric hospital specialized in the surgical management of BA that receives a large number of patients referred with high clinical suspicion for this condition. Consequently, the study population was highly selected and may not reflect the distribution of etiologies observed in broader neonatal cholestasis cohorts.

Kasai portoenterostomy remains the standard treatment and should ideally be performed before 60 days of age, as delayed intervention is associated with worse outcomes [2, 12]. Previous studies have shown that surgery performed before 50 days results in jaundice clearance in 55–60% of cases, with native liver survival of approximately 50% at five years and overall survival rates approaching 90% [18, 19]. In addition to clinical outcomes, delayed diagnosis is also associated with increased healthcare costs, longer hospital stays, higher complication rates, and earlier need for liver transplantation [13, 14]. Age at surgery remains the only potentially modifiable factor influencing outcomes [20]. Therefore, early diagnosis is critical not only for improving prognosis but also for reducing healthcare costs.

In Brazil, the mean age at diagnosis and surgical intervention remains relatively late, at approximately 90 days (12 weeks) [21]. In contrast, recent data from the Japanese Biliary Atresia Registry report a mean age at surgery of 60.6 days, while centralized care models in the United Kingdom have achieved intervention at around 50 days [19, 22]. These differences highlight the importance of early referral, structured diagnostic pathways, and healthcare system organization in ensuring timely access to Kasai portoenterostomy, a key determinant of native liver survival [4, 19, 20]. In a country of continental dimensions such as Brazil, the lack of centralized care and limited multicenter data may contribute to diagnostic delays and hinder a comprehensive understanding of national outcomes.

In our cohort, the mean age at diagnosis and surgical intervention was approximately 13 weeks, while the mean age at US evaluation was approximately 11 weeks, confirming delayed diagnostic evaluation. This delay is multifactorial and include difficulties in distinguishing pathological jaundice from physiological jaundice, under-recognition of pale stools—particularly among first-time caregivers—and delays in referral by primary healthcare providers [23]. These findings reinforce the importance of educational strategies and structured referral protocols aimed at improving early recognition of neonatal cholestasis.

The relatively advanced age of the patients included in our cohort represents an important limitation of the study. Therefore, our findings should not be interpreted as evidence that the same diagnostic performance would necessarily be reproduced in infants evaluated before 60 days of life, particularly those younger than 45 days.

Serum GGT levels proved to be a useful and accessible adjunctive biomarker. Patients with BA demonstrated significantly higher GGT levels compared to non-BA cases (607 U/L vs. 243 U/L). ROC curve analysis identified a cutoff value of 311 U/L, demonstrated good diagnostic performance, with moderate sensitivity of 82.2% and specificity of 68.2%. These findings are consistent with previous studies proposing cutoff values around 300 U/L and support the role of GGT as an adjunctive biomarker in the diagnostic evaluation of neonatal cholestasis, particularly in resource-limited settings [24–26]. 

Abdominal ultrasonography is the first-line imaging modality for neonatal cholestasis due to its non-invasive nature, widespread availability, and role in guiding surgical decisions [8]. Well-established ultrasonographic features, such as gallbladder abnormalities and the triangular cord sign, have been identified as highly specific predictors of BA in previous studies [8, 27, 28]. These biomarkers were selected due to their clinical relevance and reproducibility.

In our cohort, at least one ultrasonographic biomarker was identified in all confirmed BA cases, resulting in high sensitivity (100%) and specificity (86%), and overall accuracy (96%). Furthermore, no patient with BA failed to undergo surgical exploration. Notably, logistic regression identified irregular gallbladder wall as a strong predictor of BA (OR 284; 95% CI: 26–10,830). However, these findings should be interpreted considering the tertiary referral profile of the institution and the fact that all examinations were performed and reviewed by experienced pediatric radiologists. The reproducibility of these findings may therefore vary according to operator experience and local imaging expertise, particularly in primary or secondary healthcare settings.

Three false-positive ultrasonographic cases were observed, corresponding to patients with bile duct hypoplasia and neonatal hepatitis. This finding reinforces the potential overlap of ultrasonographic findings among different causes of neonatal cholestasis and highlights the importance of interpreting imaging results within the broader clinical and laboratory context.

The combination of US findings with elevated GGT levels (> 311 U/L) improved diagnostic specificity in our cohort (95.5%). These findings suggest the use of a combined protocol as a reliable, non-invasive, and widely applicable screening strategy to support referral and diagnostic evaluation in specialized centers, particularly in settings with limited access to advanced diagnostic methods.

Despite these promising results, some limitations should be acknowledged. The retrospective single-center design and relatively small sample size may limit external validity. In addition, the study population represented a highly selected tertiary referral cohort with a disproportionately high prevalence of BA. Important causes of neonatal cholestasis, including alpha-1 antitrypsin deficiency, PFIC, and hypopituitarism, were not represented in our sample. Furthermore, all ultrasonographic examinations were performed by experienced pediatric radiologists using standardized institutional protocols, which may limit reproducibility in less specialized centers.

Despite these limitations, the present study demonstrates that ultrasonographic biomarkers, particularly when combined with serum GGT levels, demonstrated excellent diagnostic value, offering a low-cost, accessible, and non-invasive strategy that does not require sedation. It may contribute to the diagnostic evaluation of infants with suspected BA in specialized settings. In resource-limited regions, these accessible and non-invasive tools may assist in identifying patients requiring prompt referral for further evaluation.

**Fig. 5:**
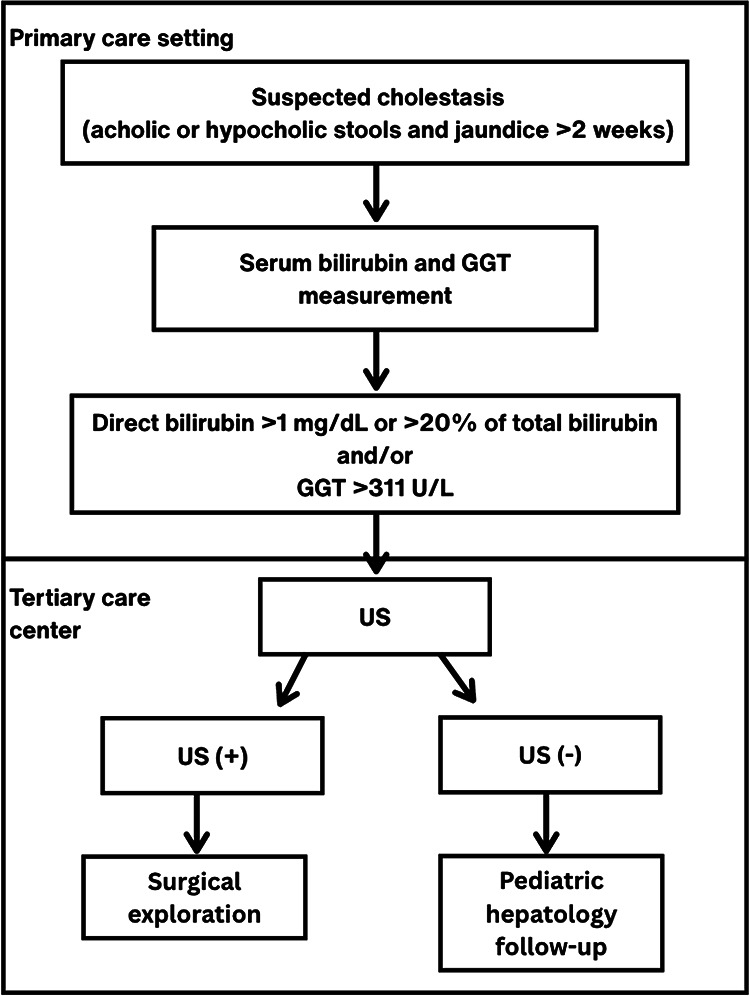
Flowchart for the evaluation of infants with suspected neonatal cholestasis. GGT, gamma-glutamyl transferase; US, ultrasonography

A proposed diagnostic protocol includes the measurement of serum bilirubin and GGT levels in primary care settings in infants with suspected cholestasis (acholic or hypocholic stools and jaundice beyond two weeks of age). Infants with direct bilirubin levels greater than 1 mg/dL and/or GGT levels above 311 U/L should be promptly referred to tertiary centers for abdominal ultrasonography. Cases with positive ultrasonographic findings should undergo urgent surgical exploration of the biliary tract (Fig. 5).

The implementation of structured diagnostic protocols incorporating these tools into the evaluation of neonatal cholestasis, together with educational initiatives directed toward primary healthcare professionals, may help reduce delays in referral and improve access to specialized care. Earlier recognition and referral may contribute to reducing delays in Kasai portoenterostomy and potentially improve clinical outcomes. However, further prospective multicenter studies involving broader and younger patient populations are necessary to validate these findings and determine the reproducibility of this diagnostic approach in different clinical settings.

In conclusion, BA remains the leading cause of neonatal cholestasis, and delayed diagnosis continues to adversely affect surgical outcomes. In this tertiary referral cohort, abdominal ultrasonography, alone or combined with serum GGT levels, demonstrated high diagnostic performance for the evaluation of BA. However, these findings should be interpreted considering the operator-dependent nature of ultrasonography, the tertiary referral profile of the institution, and the relatively late age at diagnosis observed in this population. Further prospective multicenter studies are necessary to validate these findings in broader and younger patient populations.

## Data Availability

The datasets generated and/or analyzed during the current study are not publicly available due to patient confidentiality and institutional restrictions but are available from the corresponding author on reasonable request.
